# P26 Infection or inflammation? The rheumatologist’s conundrum

**DOI:** 10.1093/rap/rkae117.057

**Published:** 2024-11-05

**Authors:** Mariam Al-Attar, Lee-Suan Teh

**Affiliations:** Royal Blackburn Hospital, Blackburn, United Kingdom; Royal Blackburn Hospital, Blackburn, United Kingdom

## Abstract

**Introduction:**

Differentiating infection from inflammation is a common challenge for the rheumatologist. Whilst it is sometimes considered safer to treat for a presumed infection as a primary strategy, or to treat for both infection and inflammation, this can be difficult when the patient is critically unwell. This report describes a patient with an unknown cause of deterioration, and summarises key learning points and reflections which can be taken from the case.

**Case description:**

A 74-year-old female patient presented to her GP with shoulder pain and a swollen right wrist. She had a background of polymyalgia rheumatica (PMR) diagnosed by her GP 15 months earlier and was on a tapering course of prednisolone. She was otherwise well. Bloods at this time revealed CRP 89, ESR 56, RF 33. The GP increased the prednisolone dose to 10 mg. One week later, the patient presented to the emergency department with worsening right wrist pain, new weakness and numbness to her right hand, and a petechial rash. Bloods demonstrated CRP 318; ESR>109, Hb 95. She was noted to have significant right-sided MCP/wrist swelling, along with numbness in in digits 1-3. The working diagnosis was new onset rheumatoid arthritis (RA) with carpal tunnel syndrome secondary to synovitis. Her prednisolone dose was increased to 15 mg. Given the petechial rash, a full immunology screen was sent.

Her immunology screen subsequently demonstrated cANCA positivity with PR3>166. Her CRP remained high at 264 and her ESR was 100. In this context, her neurological symptoms were now felt to represent median mononeuritis. She received IV methylprednisolone with resultant improvement of symptoms and CRP falling to 30. However, prior to receiving cyclophosphamide, she developed difficulty breathing and high oxygen requirements. Her CRP rose to 291 and chest X-ray showed bilateral, widespread infiltrates. She had no haemoptysis, but her Hb dropped to 67. She was started on IV antibiotics, but her respiratory condition continued to deteriorate and she required intubation. Pulmonary vasculitis/haemorrhage was considered, but it was felt to be too risky to immunosuppress further given the possibility of infection. She was given plasmapheresis alongside 40 mg prednisolone and broad-spectrum antibiotic therapy, but CRP continued to rise (peaking at 439) and her oxygen requirements continued to increase. On day 23 of admission, she unfortunately passed away.

**Discussion:**

This report describes a patient who was diagnosed with cANCA vasculitis and had a good response to IV methylprednisolone, with almost full resolution of symptoms. However, there was a delay in her receiving cyclophosphamide and, in this time period, she deteriorated from a respiratory perspective. The timing and presentation of this deterioration made it particularly difficult to diagnose; following methylprednisolone, she was in a relatively immunosuppressed state, pre-disposing her to infection. However, she had also not yet received cyclophosphamide, meaning her vasculitis was potentially not yet adequately treated. Haemoptysis may have made the diagnosis clearer but it was not present. The high CRP and diffuse infiltrates on the X-ray were both unhelpful in determining the aetiology of the deterioration. She was deemed too unstable for a broncho-alveolar lavage by the intensive care team. The respiratory/radiology teams advised that a CT would not help in differentiating infection from inflammation; however, a subsequent review of the literature revealed that a CT pulmonary angiogram may sometimes be helpful in diagnosing a pulmonary haemorrhage. The microbiology team recommended procalcitonin to be tested, but a result for this was never received.

It is interesting that this patient had a previous diagnosis of PMR. ANCA vasculitis can be misdiagnosed as PMR, particularly when patients are older and present with milder, more constitutional symptoms. In retrospect, her CRP on diagnosis of PMR was 130, and this should have been considered a red flag and prompted referral to secondary care. It is likely that her vasculitis symptoms were then masked by the low-dose steroids, and flared once her steroids were tapered. Finally, although compression (carpal tunnel syndrome) is the most common cause of median mononeuritis, vasculitis is an important non-compressive cause. Neurophysiology tests can help distinguish compressive versus non-compressive causes of mononeuritis.

**Key learning points:**

Infection or inflammation:

• Haemoptysis may be absent in a third of patients with pulmonary haemorrhage.

• CT pulmonary angiogram can be considered to identify pulmonary haemorrhage where alveolar lavage, lung biopsy and gas transfer testing are not possible.

• Procalcitonin is a useful blood test for identifying severe infections but in some hospital Trusts may take a long time to return a result, limiting use in an acutely unwell patient.

• It is important to consider the timing of a patient’s deterioration, the presence of fevers and relative immunosuppressive state; however, differentiating infection from inflammation remains a difficult challenge.

• A collaborative, multi-disciplinary approach is recommended in the acutely unwell rheumatology patient with a suspected infection, and relevant teams should always be involved e.g. intensive care, respiratory, radiology, microbiology.

Wider learning points:

• ANCA vasculitis can be misdiagnosed as PMR, particularly when patients are older and present with milder, more constitutional symptoms. However, a very high CRP should prompt consideration of wider diagnoses.

• Vasculitis is an important cause of non-compressive mononeuropathy. We should exercise caution with immediately labelling a median mononeuritis as carpal tunnel syndrome, as this potentially prematurely ends the diagnostic thought process and may lead to misdiagnosis.

